# Relationship between spleen size and exercise tolerance in advanced heart failure patients with a left ventricular assist device

**DOI:** 10.1186/s13104-022-05939-y

**Published:** 2022-02-10

**Authors:** Hiroaki Hiraiwa, Takahiro Okumura, Akinori Sawamura, Takashi Araki, Takashi Mizutani, Shingo Kazama, Yuki Kimura, Naoki Shibata, Hideo Oishi, Tasuku Kuwayama, Toru Kondo, Kenji Furusawa, Ryota Morimoto, Takuji Adachi, Sumio Yamada, Masato Mutsuga, Akihiko Usui, Toyoaki Murohara

**Affiliations:** 1 Department of Cardiology, Nagoya University Graduate School of Medicine, 65 Tsurumai-cho, Showa-ku, Nagoya, 466-8550 Japan; 2 Department of Cardiology, Ichinomiya Municipal Hospital, 2-2-22 Bunkyo, Ichinomiya, 491-8558, Japan; 3 Department of Integrated Health Sciences, Nagoya University Graduate School of Medicine, 1-1-20 Daiko-minami, Higashi-ku, Nagoya, 461-8673 Japan; 4 Department of Cardiac Surgery, Nagoya University Graduate School of Medicine, 65 Tsurumai-cho, Showa-ku, Nagoya, 466-8550 Japan

**Keywords:** Spleen volume, Exercise tolerance, Advanced heart failure, Left ventricular assist device, Structural equation modeling

## Abstract

**Objective:**

Spleen volume increases in patients with advanced heart failure (HF) after left ventricular assist device (LVAD) implantation. However, the relationship between spleen volume and exercise tolerance (peak oxygen consumption [VO_2_]) in these patients remains unknown. In this exploratory study, we enrolled 27 patients with HF using a LVAD (median age: 46 years). Patients underwent blood testing, echocardiography, right heart catheterization, computed tomography (CT), and cardiopulmonary exercise testing. Spleen size was measured using CT volumetry, and the correlations/causal relationships of factors affecting peak VO_2_ were identified using structural equation modeling.

**Results:**

The median spleen volume was 190.0 mL, and peak VO_2_ was 13.2 mL/kg/min. The factors affecting peak VO_2_ were peak heart rate (HR; β = 0.402, *P* = .015), pulmonary capillary wedge pressure (PCWP; β =  − 0.698, *P* = .014), right ventricular stroke work index (β = 0.533, *P* = .001), blood hemoglobin concentration (β = 0.359, *P* = .007), and spleen volume (β = 0.215, *P* = .041). Spleen volume correlated with peak HR, PCWP, and hemoglobin concentration, reflecting sympathetic activity, cardiac preload, and oxygen-carrying capacity, respectively, and was thus related to peak VO_2_. These results suggest an association between spleen volume and exercise tolerance in advanced HF.

**Supplementary Information:**

The online version contains supplementary material available at 10.1186/s13104-022-05939-y.

## Introduction

Associations between the heart and other organs have been studied to better understand heart failure (HF) pathophysiology [1]. The spleen is a highly vascular organ that pools 200–250 mL of blood [2]. It interacts with the kidneys and mesenteric vascular bed via neural and hormonal regulation to regulate circulating blood volume and pressure [3]. In patients with HF, the spleen regulates the balance between stressed and unstressed volumes [4]. Thus, the spleen and heart are considered to interact; however, little is known about the heart–spleen axis in HF.

Normally, the spleen contracts during circulatory insufficiency, which mobilizes stored blood into the systemic circulation to increase blood volume and aids activity in healthy people [5]. In patients with advanced HF, we previously reported a 1.4-fold increase in spleen volume after left ventricular assist device (LVAD) implantation, which was associated with an improvement in hemodynamics [6]. Additionally, spleen volume was associated with pulsatility index, reflecting cardiac preload in advanced HF patients with LVADs [7].

However, despite the observed increase in spleen volume and the improvement in hemodynamics in patients with HF after LVAD implantation [6], another study reported no improvement in exercise tolerance in these patients during cardiac rehabilitiation [8]. Our group has previously shown that right heart catheter indices at rest correlate with exercise tolerance in LVAD patients [9]. As such, resting spleen volume reflects resting right heart catheter parameters, and therefore, its relationship to exercise tolerance is a valid subject of investigation. There are many factors suggested to influence exercise tolerance, including native cardiac characteristics, including right ventricular (RV) function; non-cardiac peripheral parameters, including anemia; obesity; and general deconditioning with low muscle mass [8, 10–14]. In HF patients with LVADs, LVAD pump speed also influences exercise tolerance. However, no studies have examined the association between spleen volume and exercise tolerance in patients with LVADs. This will broaden our understanding of hemodynamics and the factors that influence exercise tolerance in patients with heart failure.

Peak oxygen consumption (VO_2_) is one of the most powerful measurement parameters among several indices of exercise tolerance. It is especially important in patients with severe HF, as it is also a prognostic factor. We aimed to investigate whether spleen volume is associated with peak VO_2_ (and thus exercise tolerance) in patients with advanced HF with LVADs. Here, we extend previously published research by providing preliminary insight into the relationship between spleen volume and peak VO_2_ from the perspective of the heart–spleen axis.

## Main text

### Methods

#### Study population

This study was an exploratory, observational, cross-sectional study. Forty-four patients with advanced HF underwent LVAD implantation (HeartMate II®, Abbott, Chicago, IL, USA) as a bridge to heart transplantation at Nagoya University Hospital between October 2013 and June 2019. Japanese patients were chosen because spleen volume is affected by race [15]. Of these 44 patients, the data of 27 patients (21 men; median age: 46 years [interquartile range [IQR] 37–55 years]) were available from electronic medical records for retrospective analysis. The data of 17 patients were excluded because blood test, echocardiography, right heart catheterization (RHC), computed tomography (CT), and cardiopulmonary exercise testing (CPET) data were not available simultaneously. All patients were followed up by expert cardiologists with optimal medical therapy according to current guidelines for HF treatment [16, 17]. The details of drug therapy are shown in Table 1. Notably, at the time of this study, angiotensin receptor-neprilysin inhibitors and sodium glucose cotransporter-2 inhibitors were not available for clinical use in Japan; thus, these drugs are not included in Table 1. All patients were hospitalized at our institution for advanced HF and were scheduled to undergo LVAD implantation, which was followed by cardiac rehabilitation. Patients underwent low-to-moderate-intensity interval strength training and sustained exercise with a Borg scale of ≤ 13 on a bicycle ergometer [18, 19].

**Table 1:** Patients’ demographic and clinical characteristics

	All patients (n = 27)
Age, years	46 (37–55)
Male	21 (78)
BSA, m^2^	1.67 (1.59–1.76)
Etiology of heart failure	
Dilated cardiomyopathy	16 (59)
Ischemic cardiomyopathy	7 (26)
Dilated phase of hypertrophic cardiomyopathy	1 (3.7)
Post-myocarditis dilated cardiomyopathy	1 (3.7)
Isolated cardiac sarcoidosis	1 (3.7)
Lupus myocarditis	1 (3.7)
Comorbidities	
Hypertension	7 (26)
Diabetes mellitus	5 (19)
Dyslipidemia	7 (26)
Atrial fibrillation	0 (0)
COPD	0 (0)
Medical therapy	
ACEis/ARBs	21 (78)
Beta-blockers	24 (89)
MRAs	23 (85)
Loop diuretics	17 (63)
Laboratory measurements	
Hemoglobin, g/dL	12.1 (10.6–13.4)
Sodium, mEq/L	139 (137–140)
Creatinine, mg/dL	0.82 (0.74–0.98)
BNP, pg/mL	73.8 (51.9–165.8)
hs-CRP, mg/dL	0.17 (0.09–0.43)
Echocardiography	
LVDD, mm	56.8 (47.5–64.1)
LVEF, %	23.2 (12.0–34.1)
E/e'	13.7 (8.1–17.3)
Moderate or severe mitral regurgitation	2 (7.4)
Cardiac catheterization	
HR, beats/min	75 (69–81)
Mean ABP, mmHg	76 (72–81)
RAP, mmHg	6 (3–8)
Mean PAP, mmHg	16 (13–18)
PCWP, mmHg	9 (5–10)
Total CO, L/min	4.6 (4.1–5.3)
RVSWI, g m/m^2^/beat	4.1 (3.5–6.0)
Cardiopulmonary exercise testing	
Exercise time, min	7.0 (5.5–7.5)
Workload, watts	73 (59–83)
Rest HR, beats/min	76 (71–86)
Peak HR, beats/min	130 (108–152)
Rest SBP, mmHg	89 (81–94)
Peak SBP, mmHg	107 (95–119)
Peak VO_2_, mL/kg/min	13.2 (11.8–16.3)
Peak RER	1.30 (1.21–1.41)
CT volumetry	
Spleen volume, mL	190 (148–221)
LVAD parameters	
Pump speed, rpm	8800 (8600–9200)
Pump power, watts	6.0 (5.4–6.4)
Pump flow, L/min	4.9 (4.3–5.4)
Pulsatility index	5.6 (4.2–6.0)

Data are presented as median (interquartile range) or n (%)

ABP: aortic blood pressure; ACEis: angiotensin-converting enzyme inhibitors; ARB: angiotensin receptor II blocker; BNP: brain natriuretic peptide; BSA: body surface area; CO: cardiac output; COPD: chronic obstructive pulmonary disease; CT: computed tomography; E/e': ratio of early transmitral flow velocity to early diastolic mitral annular velocity; HR: heart rate; hs-CRP: high-sensitivity C-reactive protein; LVAD: left ventricular assist device; LVDD: left ventricular end-diastolic dimension; LVEF: left ventricular ejection fraction; MRAs: mineralocorticoid receptor antagonists; PAP: pulmonary artery pressure; PCWP: pulmonary capillary wedge pressure; RAP: right atrial pressure; RER: respiratory exchange ratio; rpm: revolutions per minute; RVSWI: right ventricular stroke work index; SBP: systolic blood pressure; VO_2_: oxygen consumption

Blood tests, echocardiography, RHC, CT, and CPET were performed on the same day 6 months after LVAD implantation under hemodynamically stable conditions. The ethics review board of Nagoya University Hospital approved the study protocol (approval number: 2017-0281), which was conducted according to the 1975 Helsinki Declaration. All patients provided written informed consent.

#### Measurement of spleen volume using CT

**Fig. 1:**
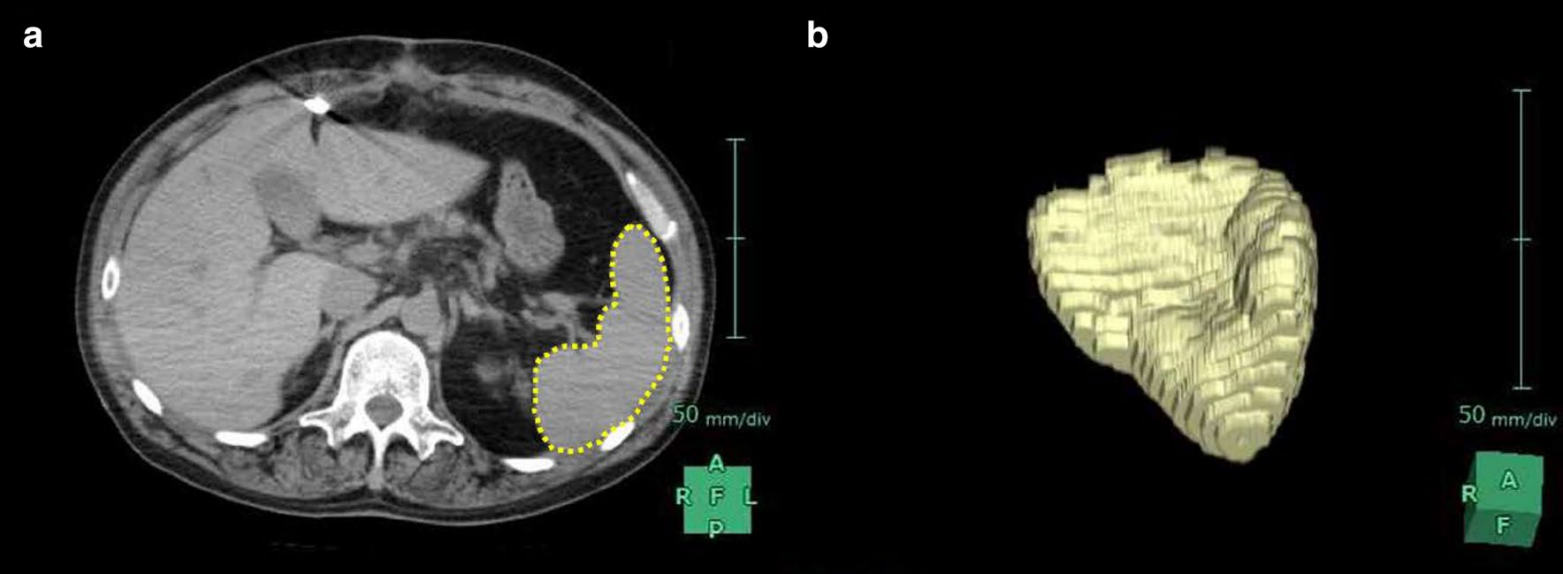
Abdominal computed tomography axial-view image from a patient with a left ventricular assist device. The spleen is surrounded by a yellow dotted line (**a**). Three-dimensional images were obtained by outlining the spleen (**b**)

All CT examinations were performed using a 64-row multidetector CT system (Aquilion® Prime SP; Canon Medical Systems Corporation, Otawara, Japan). Abdominal CT images were obtained from all patients using 1.0- or 5.0-mm slices, including non-enhanced and contrast-enhanced images. Spleen volume was measured using CT volumetry by manually drawing the outline of the spleen in three dimensions using a three-dimensional image analysis system (Synapse Vincent® version 4.3; FUJIFILM Corporation, Tokyo, Japan) (Fig. 1). Two observers, a cardiologist and a radiologist, twice conducted all measurements in a blinded fashion, and the mean values were analyzed.

#### RHC

All patients underwent RHC to assess hemodynamic status after LVAD implantation. Heart rate (HR), aortic blood pressure, right atrial pressure (RAP), pulmonary arterial pressure (PAP), and pulmonary capillary wedge pressure (PCWP) were measured. Total cardiac output (CO) was determined using the Fick method. RV stroke work index (RVSWI) was calculated using the following equation: RVSWI = (stroke volume ÷ body surface area [BSA]) × (mean PAP − mean RAP) × 0.0136 [20].

#### CPET

CPET was performed using a cycle ergometer with breath-by-breath respiratory gas measurements using a computerized metabolic cart (AE-310S; Minato Medical Science, Osaka, Japan). After a 3-min rest on the ergometer, exercise began with a 3-min warm-up at 20 watts and 50 repetitions/minute, followed by a 10-W ramp loading every minute. The electrocardiogram, HR, peak VO_2_, and carbon dioxide production were continuously monitored, and non-invasive blood pressure was measured every minute during exercise. The test was terminated when the patient showed (1) maximal volitional fatigue or (2) VO_2_ leveling off. Peak oxygen pulse was calculated by dividing peak VO_2_ by peak HR to estimate peak stroke volume during exercise [21].

#### Statistical analyses

All statistical analyses were performed using SPSS statistical software (PASW® 18.0; SPSS Inc., Chicago, IL, USA). Structural equation modeling (SEM), which tests the causality for an event, was performed using Stata software (Stata® 15.0; StataCorp., College Station, TX, USA). With SEM, β is the path coefficient, which represents the strength between variable items [22]. The higher the β value, the stronger the relationship between the variable items. The advantage of SEM is that it can quantify the strength of the relationship between variables. For this reason, we did not use Pearson’s correlation coefficient in this study. Continuous variables are expressed as median (IQR). Categorical variables are expressed as number (%). The correlations and causal relationships of factors that contribute to peak VO_2_ were investigated using SEM. A *P* value of < 0.05 were considered statistically significant.

### Results

Patients’ characteristics are presented in Table 1. The most frequent HF etiology was idiopathic dilated cardiomyopathy. Most patients continued optimal medical treatment for HF. However, medications were not included in SEM because we did not identify any significant confounding status in the correlation analysis. All patients remained stable with LVAD support. LVAD pump speed ranged from 8400 to 9800 rpm. Median spleen volume was 190.0 mL (148.0–221.0 mL), and spleen volume was < 200 mL in 59% of patients (Additional file 1: Fig. S1).

**Fig. 2:**
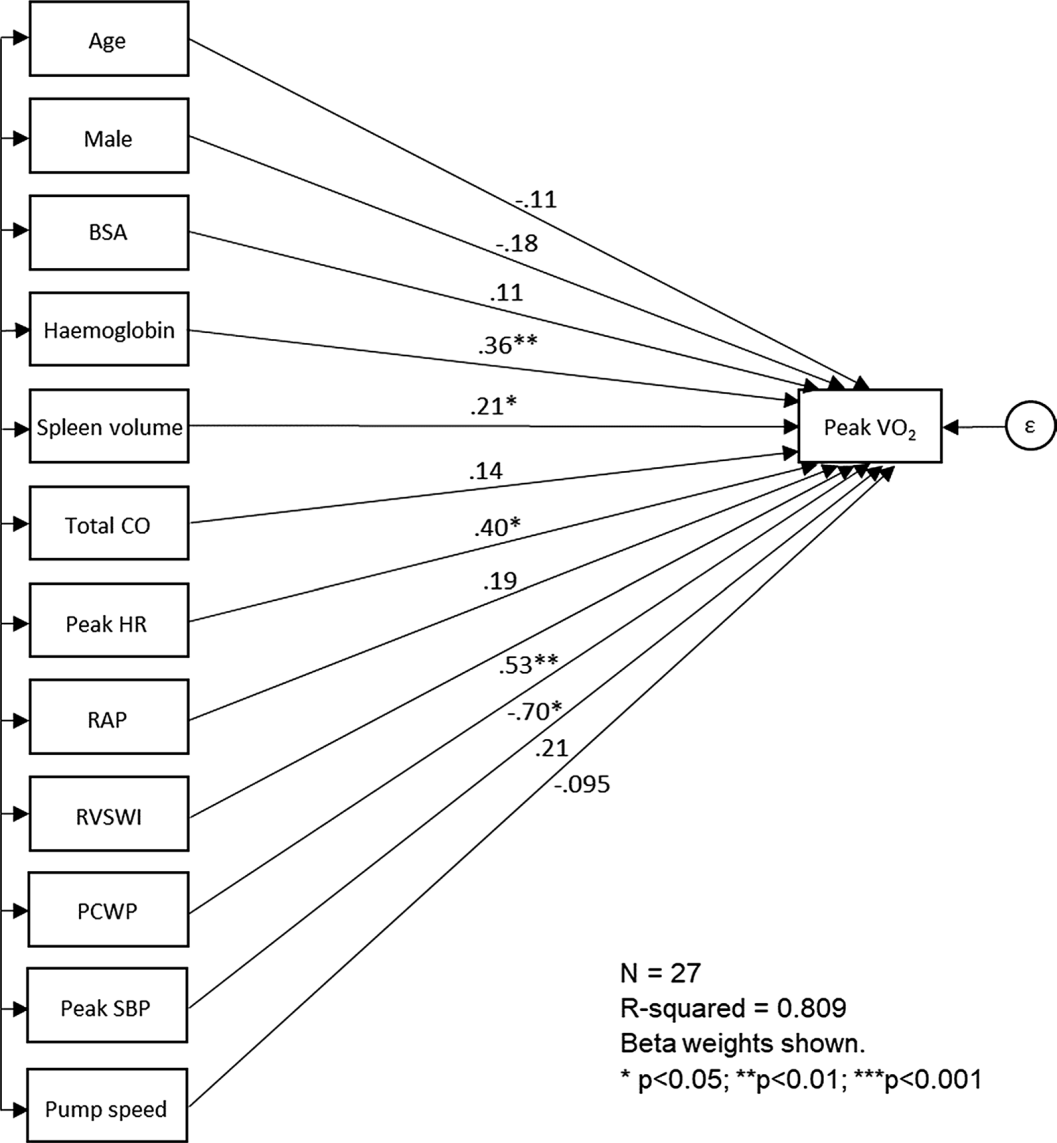
Diagram of structural equation modeling to represent correlations/causal relationships among factors that influence peak VO_2_. BSA, body surface area; CO; cardiac output; HR: heart rate; PCWP: pulmonary capillary wedge pressure; RAP: right atrial pressure; RVSWI: right ventricular stroke work index; SBP: systolic blood pressure; peak VO_2_: peak oxygen consumption

With SEM, the correlations and causalities of factors that affected peak VO_2_, including age, male sex, BSA, blood hemoglobin concentration, spleen volume, total CO, peak HR, RAP, RVSWI, PCWP, peak systolic blood pressure, and pump speed (Additional file 2: Table S1 and Additional file 3: Table S2). Spleen volume was positively correlated with blood hemoglobin concentration, peak HR, RAP, and PCWP, yet negatively correlated with age. Although spleen volume was not correlated with pump speed, power, or flow, it was associated with pulsatility index (data not shown). In addition, five factors significantly affected peak VO_2_: peak HR (β = 0.402, *P* = 0.015), PCWP (β =  − 0.698, *P* = 0.014), RVSWI (β = 0.533, *P* = 0.001), blood hemoglobin concentration (β = 0.359, *P* = 0.007), and spleen volume (β = 0.215, *P* = 0.041) (Additional file 2: Table S1; Fig. 2). Moreover, spleen volume was associated with peak VO_2_ through its correlation with peak HR (β = 0.626, *P* < 0.001), PCWP (β = 0.519, *P* < 0.001), and blood hemoglobin concentration (β = 0.349, *P* = 0.047) (Additional file 2: Table S1). In contrast, there was no correlation between spleen volume and RVSWI (β = 0.222, *P* = 0.243) (Additional file 2: Table S1).

### Discussion

To our knowledge, this is the first study to demonstrate an association between spleen volume and exercise tolerance in advanced HF patients with LVADs. Although we did not show a significant direct association between spleen volume and exercise tolerance, the results of SEM show that spleen volume is indirectly associated with peak VO_2_ through its relationship with peak HR, PCWP, and blood hemoglobin concentration. It is possible that a direct relationship exists between spleen volume and peak VO_2_, but that it was not detected because of limitations in our statistical method, such as the small sample size and the large number of factors included in the SEM. Despite this, our findings of an indirect association may offer new insights into the heart–spleen axis relative to the exercise tolerance of advanced HF patients with LVADs.

A previous report described that spleen volume in adults is affected by age, sex, and height. The healthy spleen is usually 12 cm long, 7 cm broad, and 3–4 cm wide [23]. Spleen volume in patients with advanced HF with LVADs in this study was approximately the same or slightly greater than that reported in healthy Japanese individuals in a previous study [15]. Therefore, we used age, sex, and BSA to analyze the association between spleen volume and peak VO_2_ using SEM.

Previous studies on exercise tolerance in LVAD patients have reported that age, LVAD pump speed, RV function, and extra-cardiac factors, such as anemia, low skeletal muscle mass, and exercise training, may determine peak VO_2_ [8, 10–14]. However, in this study, with SEM, peak VO_2_ was not associated with age (β =  − 0.108, *P* = 0.420) or pump speed (β =  − 0.095, *P* = 0.474), but it was associated with RVSWI (β = 0.533, *P* = 0.001) (Additional file 2: Table S1). The reason for these findings may be related to the specificity of the study population, which was small in size and comprised a high proportion of males. RVSWI, which is an index of RV function, was strongly associated with peak VO_2_, which is consistent with previous studies [8, 9]. One study reported that peak CO is best correlated with peak VO_2_ in HF patients with LVADs [24]. We did not evaluate the parameters of RHC directly during exercise. However, we previously reported that hemodynamic parameters of RHC at rest can predict peak VO_2_ in patients with LVADs [9]. Furthermore, this is profitable in terms of patient safety to avoid performing catheterization during exercise.

In this study, spleen volume was associated with peak HR. There was also a positive correlation between spleen volume and resting HR (data not shown). In severe HF patients before LVAD implantation, spleen volume was negatively correlated with systemic vascular resistance (SVR) under conditions of increased sympathetic nerve activity. Conversely, there was no correlation between spleen volume and SVR in patients with improved hemodynamics and sympathetic hyperactivity after LVAD implantation [6]. No previous reports have examined the relationship between spleen volume and HR in patients with HF. We consider one mechanism to explain this relationship; a large spleen reflects normal and stable sympathetic nerve activity at rest, and sympathetic nerve activity is elevated during exercise.

In this study, spleen volume was positively correlated with PCWP, and PCWP was negatively associated with peak VO_2_ (Additional file 2: Table S1). The correlation between spleen volume and PCWP has been reported in patients with LVADs previously [7]. Additionally, in HF patients with LVADs, a low PCWP maintained peak VO_2_, which is consistent with previous studies [8, 10–14]. As a mechanism, PCWP is thought to reflect left ventricular preload and stressed volume status. Spleen volume and RAP also correlated positively, and RAP is thought to reflect RV preload. RVSWI is a determinant of exercise tolerance in LVAD patients [8]. In the present study, there was no significant correlation between spleen volume and RVSWI, but RVSWI had a direct effect on peak VO_2_, which is consistent with previous studies [8, 9].

Furthermore, spleen volume and peak VO_2_ were positively correlated with blood hemoglobin concentration. An association between peak VO_2_ and blood hemoglobin concentration was revealed using the Fick equation, because blood hemoglobin concentration is an important indicator of oxygen-carrying capacity. Temporary hypoxia during exercise, breath-holding during diving, and high-altitude activity lead to spleen contraction and a significant increase in blood hemoglobin concentration [25–27]. The spleen contracts in response to adrenergic stimulation and responds by mobilizing stored blood, releasing erythrocytes into the systemic circulation [5, 28–30]. In addition, spleen volume in people who habitually engage in diving is changed as an environmental adaptation [31], and the spleen has a profound effect on exercise tolerance. These findings suggest that blood hemoglobin concentration is related to spleen volume, and the spleen contributes to peak VO_2_ in severe HF patients with LVADs by increasing the oxygen-carrying capacity of the blood.

### Conclusions

In this study, spleen size was associated with exercise capacity in advanced HF patients with LVADs through its relationship with HR, PCWP, and blood hemoglobin concentration, reflecting sympathetic activity, cardiac preload, and oxygen-carrying capacity. These preliminary findings suggest that spleen volume may thus be worthy of investigation as an indicator of hemodynamics and exercise tolerance given its association with peak VO_2_ in advanced HF patients with LVADs.

## Limitations

The strengths of this preliminary study include the collection of data on multiple variables to allow for a comprehensive exploratory analysis using SEM. The use of SEM is another strength of this study because SEM tests the causality for an event and can quantify the strength of the relationship between variables. Moreover, we report a previously unobserved phenomenon with potential clinical relevance that warrants further investigation. In terms of the clinical implications of this study, we believe that measuring blood hemoglobin concentration and spleen volume could be useful in the clinic to assess exercise tolerance in LVAD patients with heart failure.

However, this study also has several limitations that should be noted. First, the sample size was small, which may have prevented us from detecting a direct association between spleen volume and peak VO_2_, and all patients were from a single center. Large-sample studies performed at multiple centers are required to clarify and generalize our findings.

Second, it is possible that many confounding factors were not evaluated. For example, peripheral skeletal muscle function was not examined as an influencer of arteriovenous oxygen difference, although all patients underwent cardiac rehabilitation. Therefore, the relationship between spleen volume and exercise tolerance in LVAD patients might not have been sufficiently addressed.

Third, CT to measure spleen volume results in radiation exposure. Therefore, further studies using non-invasive measurements to evaluate spleen volume, such as abdominal ultrasonography, are needed.

Fourth, the study lacked a control group, which is important in observational studies for data validation. Nonetheless, we believe our study has value as a preliminary indication that spleen size is associated with exercise tolerance in patients with advanced HF, warranting further study.

Finally, the small number of patients included in this preliminary study meant that the analysis was underpowered. We recommend that future studies include a sufficiently large sample size to achieve adequate statistical power for analysis.

## Supplementary Information

**Additional file 1: Figure S1.** Histogram of spleen volume in all patients.

**Additional file 2: Table S1.** Structural equation modeling to represent correlations or causal relationships among factors that influence peak VO_2_.

**Additional file 3: Table S2.** Structural equation modeling to represent correlations among parameters associated with peak VO_2_.

## Data Availability

The datasets supporting the conclusions of this article are available from the corresponding author upon reasonable request.

## Abbreviations

BSA: Body surface area
CO: Cardiac output
CT: Computed tomography
HF: Heart failure
HR: Heart rate
LVAD: Left ventricular assist device
PAP: Pulmonary arterial pressure
PCWP: Pulmonary capillary wedge pressure
RAP: Right atrial pressure
RV: Right ventricular
RVSWI: Right ventricular stroke work index
VO_2_: Oxygen consumption
