# Supplementary figures and images for: Relationship between spleen size and exercise tolerance in advanced heart failure patients with a left ventricular assist device
Source: BMC Res Notes. 2022 Feb 10;15:40. doi: 10.1186/s13104-022-05939-y (PMC8832641; doi:10.1186/s13104-022-05939-y)

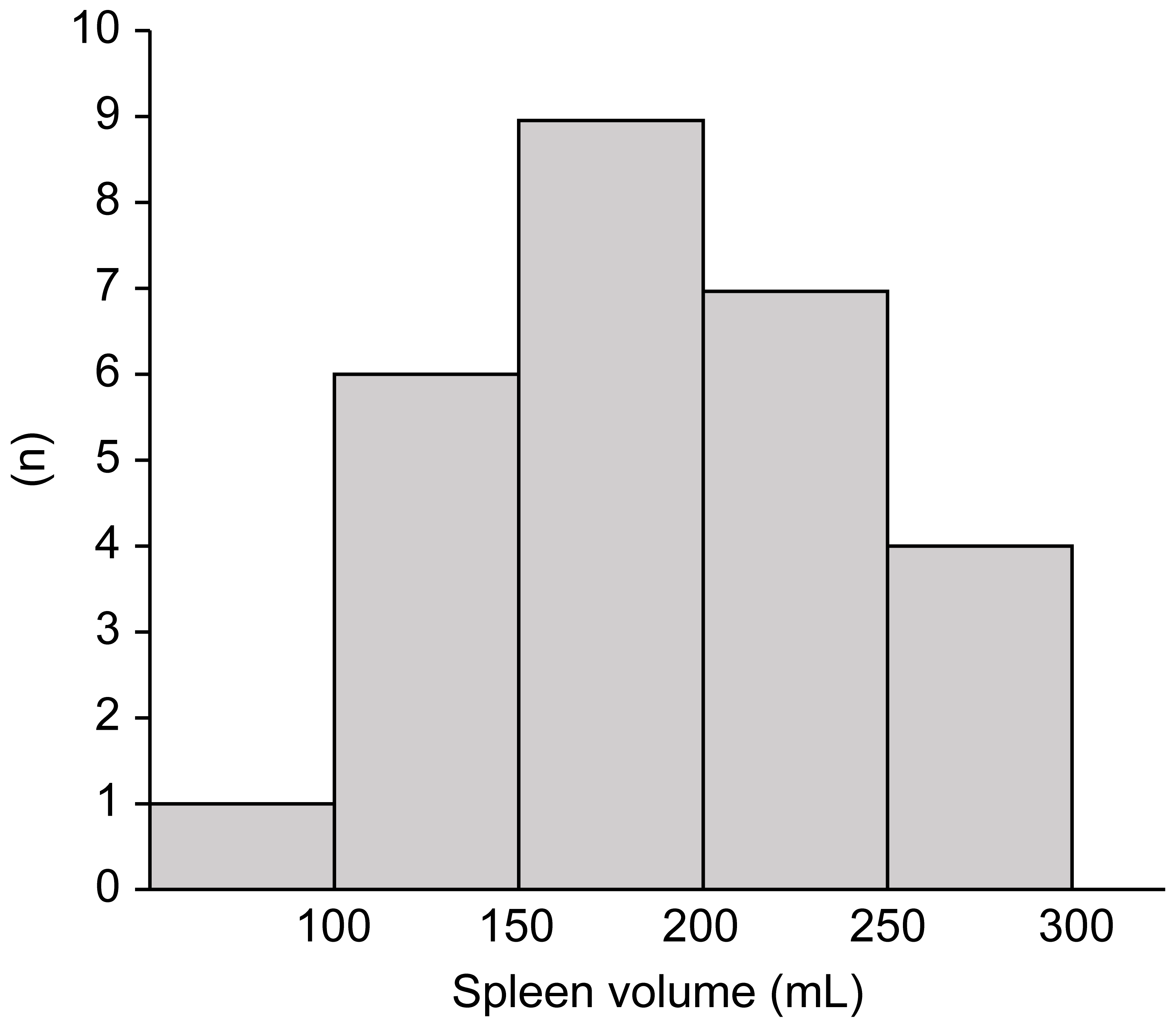

Supplement: Supplementary file 1 — Additional file 1: Figure S1. Histogram of spleen volume in all patients. [file 13104_2022_5939_MOESM1_ESM.tif]
