# Supplementary material for: Relationship between spleen size and exercise tolerance in advanced heart failure patients with a left ventricular assist device
Source: BMC Res Notes. 2022 Feb 10;15:40. doi: 10.1186/s13104-022-05939-y (PMC8832641; doi:10.1186/s13104-022-05939-y)
Supplement: Supplementary file 2 — Additional file 2: Table S1. Structural equation modeling to represent correlations or causal relationships among factors that influence peak VO2. [file 13104_2022_5939_MOESM2_ESM.doc]

**Table S1 Structural equation modeling to represent correlations or causal relationships among factors that influence peak VO_2_.**

| **Parameter relationship** | | | **Standardized coefficient　(β)** | **Standard error** | **z** | **p value** | **95% confidence interval** | |
| --- | --- | --- | --- | --- | --- | --- | --- | --- |
| Peak VO_2_ | ← | Age | −0.108 | 0.134 | −0.81 | .420 | −0.370 | 0.154 |
| Peak VO_2_ | ← | Male | −0.184 | 0.173 | −1.06 | .289 | −0.523 | 0.156 |
| Peak VO_2_ | ← | BSA | 0.113 | 0.227 | 0.50 | .618 | −0.331 | 0.557 |
| Peak VO_2_ | ← | Hemoglobin | 0.359 | 0.133 | 2.70 | .007 | 0.099 | 0.619 |
| Peak VO_2_ | ← | Spleen volume | 0.215 | 0.149 | 0.77 | .041 | −0.177 | 0.407 |
| Peak VO_2_ | ← | Total CO | 0.139 | 0.181 | 0.77 | .442 | −0.215 | 0.493 |
| Peak VO_2_ | ← | Peak HR | 0.402 | 0.165 | 2.43 | .015 | 0.078 | 0.725 |
| Peak VO_2_ | ← | RAP | 0.190 | 0.176 | 1.07 | .283 | −0.156 | 0.535 |
| Peak VO_2_ | ← | RVSWI | 0.533 | 0.165 | 3.24 | .001 | 0.211 | 0.856 |
| Peak VO_2_ | ← | PCWP | −0.698 | 0.285 | −2.45 | .014 | −1.257 | −0.140 |
| Peak VO_2_ | ← | Peak SBP | 0.211 | 0.140 | 1.50 | .132 | −0.064 | 0.486 |
| Peak VO_2_ | ← | Pump speed | −0.095 | 0.133 | −0.72 | .474 | −0.356 | 0.166 |
|  |  |  |  |  |  |  |  |  |
| Spleen volume | ↔ | Age | −0.362 | 0.174 | −2.08 | .037 | −0.703 | −0.021 |
| Spleen volume | ↔ | Male | 0.275 | 0.185 | 1.48 | .138 | −0.088 | 0.637 |
| Spleen volume | ↔ | BSA | 0.275 | 0.185 | 1.48 | .138 | −0.088 | 0.637 |
| Spleen volume | ↔ | Hemoglobin | 0.349 | 0.176 | 1.99 | .047 | 0.005 | 0.693 |
| Spleen volume | ↔ | Total CO | 0.269 | 0.186 | 1.45 | .147 | −0.095 | 0.633 |
| Spleen volume | ↔ | Peak HR | 0.626 | 0.122 | 5.14 | <.001 | 0.387 | 0.864 |
| Spleen volume | ↔ | RAP | 0.461 | 0.158 | 2.93 | .003 | 0.152 | 0.770 |
| Spleen volume | ↔ | RVSWI | 0.222 | 0.190 | 1.17 | .243 | −0.151 | 0.595 |
| Spleen volume | ↔ | PCWP | 0.519 | 0.146 | 3.55 | <.001 | 0.233 | 0.806 |
| Spleen volume | ↔ | Peak SBP | 0.184 | 0.193 | 0.95 | .342 | −0.195 | 0.562 |
| Spleen volume | ↔ | Pump speed | 0.038 | 0.200 | 0.19 | .850 | −0.354 | 0.429 |

BSA, body surface area; CO, cardiac output; HR, heart rate; PCWP, pulmonary capillary wedge pressure; RAP, right atrial pressure; RVSWI, right ventricular stroke work index; SBP, systolic blood pressure; VO_2_, oxygen consumption.
