# Supplementary material for: Relationship between spleen size and exercise tolerance in advanced heart failure patients with a left ventricular assist device
Source: BMC Res Notes. 2022 Feb 10;15:40. doi: 10.1186/s13104-022-05939-y (PMC8832641; doi:10.1186/s13104-022-05939-y)
Supplement: Supplementary file 3 — Additional file 3: Table S2. Structural equation modeling to represent correlations among parameters associated with peak VO2. [file 13104_2022_5939_MOESM3_ESM.doc]

**Table S2 Structural equation modeling to represent correlations among parameters associated with peak VO_2_.**

| **Parameter relationship** | | | **Standardized coefficient　(β)** | **Standard error** | **z** | **p value** | **95% confidence interval** | |
| --- | --- | --- | --- | --- | --- | --- | --- | --- |
| Peak HR | ↔ | Age | −0.495 | 0.151 | −3.28 | .001 | −0.791 | −0.199 |
| Peak HR | ↔ | Male | 0.096 | 0.198 | 0.49 | .627 | −0.292 | 0.485 |
| Peak HR | ↔ | BSA | 0.318 | 0.180 | 1.77 | .077 | −0.034 | 0.670 |
| Peak HR | ↔ | Hemoglobin | 0.557 | 0.138 | 4.04 | .000 | 0.286 | 0.827 |
| Peak HR | ↔ | Total CO | 0.050 | 0.200 | 0.25 | .802 | −0.341 | 0.441 |
| Peak HR | ↔ | RAP | 0.438 | 0.162 | 2.71 | .007 | 0.121 | 0.755 |
| Peak HR | ↔ | RVSWI | 0.015 | 0.200 | 0.07 | .941 | −0.377 | 0.407 |
| Peak HR | ↔ | PCWP | 0.520 | 0.146 | 3.56 | .000 | 0.234 | 0.806 |
| Peak HR | ↔ | Peak SBP | 0.338 | 0.177 | 1.91 | .057 | −0.010 | 0.685 |
| Peak HR | ↔ | Pump speed | 0.049 | 0.200 | 0.24 | .807 | −0.342 | 0.440 |
|  |  |  |  |  |  |  |  |  |
| Hemoglobin | ↔ | Age | −0.351 | 0.175 | −2.00 | .045 | −0.695 | -0.008 |
| Hemoglobin | ↔ | Male | −0.004 | 0.200 | −0.02 | .985 | −0.396 | 0.388 |
| Hemoglobin | ↔ | BSA | 0.252 | 0.187 | 1.35 | .178 | −0.115 | 0.619 |
| Hemoglobin | ↔ | RAP | −0.008 | 0.200 | −0.04 | .967 | −0.400 | 0.384 |
| Hemoglobin | ↔ | RVSWI | −0.006 | 0.200 | −0.03 | .976 | −0.398 | 0.386 |
| Hemoglobin | ↔ | PCWP | 0.208 | 0.191 | 1.09 | .277 | −0.167 | 0.583 |
| Hemoglobin | ↔ | Peak SBP | 0.233 | 0.189 | 1.23 | .218 | −0.138 | 0.604 |
| Hemoglobin | ↔ | Pump speed | −0.223 | 0.190 | −1.17 | .240 | −0.596 | 0.149 |
|  |  |  |  |  |  |  |  |  |
| PCWP | ↔ | Peak SBP | 0.363 | 0.174 | 2.09 | .036 | 0.023 | 0.704 |
| PCWP | ↔ | Pump speed | −0.009 | 0.200 | −0.04 | .965 | −0.401 | 0.383 |

BSA, body surface area; CO, cardiac output; HR, heart rate; PCWP, pulmonary capillary wedge pressure; RAP, right atrial pressure; RVSWI, right ventricular stroke work index; SBP, systolic blood pressure; VO_2_, oxygen consumption.
